# Exploring the Genetic Correlation Between Growth and Immunity Based on Summary Statistics of Genome-Wide Association Studies

**DOI:** 10.3389/fgene.2018.00393

**Published:** 2018-09-14

**Authors:** Zhe Zhang, Peipei Ma, Qiumeng Li, Qian Xiao, Hao Sun, Babatunde Shittu Olasege, Qishan Wang, Yuchun Pan

**Affiliations:** ^1^Department of Animal Science, School of Agriculture and Biology, Shanghai Jiao Tong University, Shanghai, China; ^2^Shanghai Key Laboratory of Veterinary Biotechnology, Shanghai, China

**Keywords:** GWAS, summary statistics, growth, immune, genetic correlation, pleiotropy

## Abstract

The relationship between growth and immune phenotypes has been presented in the context of physiology and energy allocation theory, but has rarely been explained genetically in humans. As more summary statistics of genome-wide association studies (GWAS) become available, it is increasingly possible to explore the genetic relationship between traits at the level of genome-wide summary statistics. In this study, publicly available summary statistics of growth and immune related traits were used to evaluate the genetic correlation coefficients between immune and growth traits, as well as the cause and effect relationship between them. In addition, pleiotropic variants and KEGG pathways were identified. As a result, we found negative correlations between birthweight and immune cell count phenotypes, a positive correlation between childhood head circumference and eosinophil counts (EO), and positive or negative correlations between childhood body mass index and immune phenotypes. Statistically significant negative effects of immune cell count phenotypes on human height, and a slight but significant negative influence of human height on allergic disease were also observed. A total of 98 genomic regions were identified as containing variants potentially related to both immunity and growth. Some variants, such as rs3184504 located in *SH2B3*, rs13107325 in *SLC39A8*, and rs1260326 located in *GCKR*, which have been identified to be pleiotropic SNPs among other traits, were found to also be related to growth and immune traits in this study. Meanwhile, the most frequent overlapping KEGG pathways between growth and immune phenotypes were autoimmune related pathways. Pleiotropic pathways such as the adipocytokine signaling pathway and *JAK-STAT* signaling pathway were also identified to be significant. The results of this study indicate the complex genetic relationship between growth and immune phenotypes, and reveal the genetic background of their correlation in the context of pleiotropy.

## Introduction

Both human growth and immune traits are influenced by inherited genetic variants (Ogata, 2006; Roederer et al., 2015). The heritability of growth traits ranges from moderate (e.g., 40% for birthweight; Johnson et al., 2011) to high (e.g., 80% for adult height; Silventoinen et al., 2003; Macgregor et al., 2006), whereas a broader range of heritability has been observed for immune traits, as they are differentially influenced by genetic and environmental factors (Mangino et al., 2017). In recent decades, studies of the associations between genotypes and phenotypes, or genome-wide association studies (GWAS), have mapped 100s of single nucleotide polymorphisms (SNPs) in association with complex traits (Donnelly, 2008; Hindorff et al., 2009; Visscher et al., 2017). For growth traits, a large number of variants relating to human height (Wood et al., 2014), obesity (Larder et al., 2017), and early growth have been identified, and have been curated by some databases such as Early Growth Genetics Consortium (EGG). Gene mapping studies have also successfully identified immunity-related traits or diseases, and nearly all major immune-mediated diseases have been studied by GWAS (Visscher et al., 2017).

Meanwhile, the increasing number of GWAS indicates the existence of underlying overlapping causal variants that play roles in multiple traits, namely pleiotropy (Visscher et al., 2017). The genetic relationships among multiple traits often result from pleiotropy of a gene and linkage disequilibrium (LD) between genes for different traits (Bolormaa et al., 2014). The former is known as biological pleiotropy, whereas the latter is a type of spurious pleiotropy (Solovieff et al., 2013). Several researches have uncovered the relationship between immune and growth traits. For example, in the context of physiology, several cytokines such as interleukin-1 (IL-1), tumor necrosis factor alpha (TNFα), and interleukin-6 (IL-6) that are released during the immune response are either growth factors (Ozaki and Leonard, 2002) or indirectly involved in the regulation of growth-related processes (Klasing, 1988). On the contrary, receptors for growth hormone (GH) and insulin-like growth factor type I (IGF-1) were found to be distributed on immunological cells (Meazza et al., 2004). The genetic relationship between growth and immunity has been primarily studied on model organisms or livestock, and this relationship has often proved to be inverse (Greer, 2008; Clapperton et al., 2009; van der Most et al., 2011).

Studies of the genetic relationship between growth and immune traits in humans have rarely been reported. This may be so as they appear to be biologically distant. However, some studies have shown that biologically unrelated traits are in fact genetically correlated. For instance, in a study of genetic correlations across human diseases and traits, height was found to be significantly associated with coronary artery disease (Bulik-Sullivan et al., 2015). Moreover, the availability of large numbers of summary statistics from GWAS has enabled increasing numbers of meta-analysis studies to explore the pleiotropy of variants, helping to elucidate the genetic relationship among traits (Han et al., 2016; Pickrell et al., 2016; Zhu et al., 2016).

The objective of this study was to employ the summary statistics of growth- and immunity-related GWAS to explore the genetic relationship between growth and immune traits, and the underlying contribution of pleiotropy across the genome to this relationship.

## Materials and Methods

### Growth and Immune Summary Statistics

A total of 15 GWAS including summary statistics of 13 growth traits and 13 immune traits were included in this study (**Table 1**). Summary statistics were selected according to the following standards: (1) non-sex-stratified European ancestry; (2) signed summary statistics; (3) without adjusting for heritable covariates. The growth summary statistics were mainly from the EGG database, height (HEIGHT) (Wood et al., 2014) in the GIANT database, together with two pediatric musculoskeletal traits, bone mineral density (BMD) and total-body lean mass (LM) (Medina-Gomez et al., 2017), distributed in GWAS Catalog database. The immune phenotypes were all from the GWAS Catalog database, and comprised a majority of immune traits belonging to the innate immune system, including a variety of immune cell count phenotypes (Astle et al., 2016). In addition, allergic disease (ALL) (Ferreira et al., 2017), asthma (ATH) (Demenais et al., 2018), and three immunity-related diseases, Crohn’s disease (CD), inflammatory bowel disease (IBD), and ulcerative colitis (UC), were also included. The summary statistics were reformatted according to the 1000 Genomes (1000G) phase 3 using script munge_sumstats.py implemented in ldsc software (URLs), as described previously (Bulik-Sullivan et al., 2015).

**Table 1:** Name, abbreviation, *P*-value threshold, and original publication for each phenotype included in this study.

Phenotype	Abbreviation	*P* threshold^a^	Publication
**Immune phenotypes**			
Any diseases	ALL	5 × 10^−8^	Ferreira et al., 2017
Asthma	ATH	5 × 10^−8^	Demenais et al., 2018
Crohn’s disease	CD	5 × 10^−8^	de Lange et al., 2017
Eosinophil counts	EO	8.31 × 10^−^9	Astle et al., 2016
Granulocyte count	GRAN	8.31 × 10^−9^	Astle et al., 2016
Inflammatory bowel disease	IBD	5 × 10^−8^	de Lange et al., 2017
Lymphocyte counts	LYMPH	8.31 × 10^−9^	Astle et al., 2016
Monocyte count	MONO	8.31 × 10^−9^	Astle et al., 2016
Myeloid white cell count	MWBC	8.31 × 10^−9^	Astle et al., 2016
Neutrophil count	NEUT	8.31 × 10^−9^	Astle et al., 2016
Ulcerative colitis	UC	5 × 10^−8^	de Lange et al., 2017
White blood cell count	WBC	8.31 × 10^−9^	Astle et al., 2016
**Growth phenotypes**			
Birth length	BL	1 × 10^−5^	van der Valk et al., 2014
Bone mineral density	BMD	5 × 10^−8^	Medina-Gomez et al., 2017
Childhood body mass index	BMI	5 × 10^−8^	Felix et al., 2015
Birthweight	BW	5 × 10^−8^	Horikoshi et al., 2016
Gestational weight gain (maternal)	GWGM	1 × 10^−5^	Warrington et al., 2018
Gestational weight gain (offspring)	GWGO	1 × 10^−5^	Warrington et al., 2018
Childhood head circumference	HC	1 × 10^−5^	Taal et al., 2012
Height	HEIGHT	5 × 10^−8^	Wood et al., 2014
Leptin levels	LEP	1 × 10^−5^	Kilpeläinen et al., 2016
Total-body lean mass	LM	1 × 10^−5^	Medina-Gomez et al., 2017
Birthweight (maternal)	MBW	5 × 10^−8^	Beaumont et al., 2018
Childhood obesity	OBESITY	5 × 10^−8^	Bradfield et al., 2012
Pubertal growth	PG	5 × 10^−8^	Cousminer et al., 2013

*^a^The significance threshold used for the GWAS for each phenotype, which was normally from the original study, but was lowered to 1 × 10^−^^5^ when the number of GWS SNPs was too low.*

### Correlation of Effect Sizes Between GWAS Summary Statistics for Immune and Growth Traits

A cross-trait LD Score regression method (Bulik-Sullivan et al., 2015) was used to evaluate the genome-wide genetic correlation between growth and immune traits. The LD score for a SNP is defined as the sum over all squared correlations between all SNPs with the focal SNP, and indicates how likely a SNP tags its neighbors affecting the phenotype. LD score regression for a single GWAS with χ^2^ statistics of SNP as a dependent variable can be used to estimate heritability. As instructed by Bulik-Sullivan et al. (2015), the traits with *Z* scores of heritabilities less than 4 were excluded in this step. When estimating genetic correlation between traits, the dependent variable of LD score regression is the product of two *Z* statistics. Unlike Mendelian randomization, which simply employs significantly associated SNPs (Davey Smith and Hemani, 2014), cross-trait LD Score regression makes use of the effects of all SNPs to estimate the correlation with the following formula:

$$E[z_{1j}z_{2j}]=\frac{\sqrt{N_{1}N_{2}}\rho_{g}}{M}l_{j}+\frac{N_{s}\rho }{\sqrt{N_{1}N_{2}}}$$

Where *Z_ij_* is the *Z* statistic for *j*th locus in study *i*, *ρ_g_* is the genetic covariance, *l_j_* is the LD Score for *j*th locus, *N_s_* is the number of overlapping individuals between studies, and ρ is the phenotypic covariance, which equals genetic covariance plus residual covariance between studies. Thus, the overlapping samples between GWAS only affect the intercept from the regression, but not the slope containing the genetic correlation between traits. In this study, we downloaded the LD Score (URLs) that had already been calculated for European ancestry using ldsc software.

### Mendelian Randomization Based on Summary Statistics of Immune and Growth Traits

To determine whether there is a cause and effect relationship between each pair of growth and immune traits and to identify the upstream causal factor and the downstream consequence, a bi-directional Generalized Summary-data based Mendelian randomization (GSMR) was performed using GSMR software (Zhu et al., 2018). GSMR belongs to the category of two-sample Mendelian randomization, but also allows bi-directional Mendelian randomization analysis (Zheng et al., 2017). This method first tests for causal associations (*b_xy_*) between a risk factor (x) and an outcome (y) based on summary statistics of each SNP (z) for x (*b_zx_*) and y (*b_zy_*), and then the *b_xy_* estimates of all the SNPs are integrated by generalized least squares. Here, pleiotropy is a potential confounding factor for GSMR, because it inflates the cause and effect relationship between exposure and outcome. Therefore, a method called HEIDI-outlier implemented in GSMR was utilized to exclude clear pleiotropic effects on the exposure and outcome phenotypes. As GSMR assumes no overlapping samples between GWAS, the pairs of growth and immune phenotypes that shared overlapping cohorts were excluded. GSMR requires independent genome-wide significant (GWS) SNPs in the analysis, which were identified based on the significance threshold. The threshold for each GWAS is listed in **Table 1** according to its original reference, except for birth length (BL), gestational weight gain (maternal) (GWGM), gestational weight gain (offspring) (GWGO), head circumference (HC), leptin levels (LEP), and LM, for which the thresholds were lowered to 1 × 10^−5^ due to the small number of GWS SNPs for these phenotypes. Then, the near-independent GWS SNPs were identified using the clumping algorithm in PLINK 1.9 (Purcell et al., 2007) for each trait [with 0.1 as cut-off for *r*^2^ in windows predefined by independent LD blocks for European ancestry (URLs)]. The allele frequency and LD information used for GSMR was from the European population in the 1000G Project. The bi-directional causation was then explored by treating growth phenotypes or immune phenotypes as exposures or outcomes alternately.

### Detection of Pleiotropy Along the Genome Between Immune and Growth Traits

A hierarchical method was used for co-localization of signals associated with immune and growth traits (Giambartolomei et al., 2014; Pickrell et al., 2016). This method estimates the regional Bayes factors for independent genomic regions along the genome for four models: (1) a genetic variant influencing trait 1 is contained in the region; (2) a genetic variant influencing trait 2 is contained in the region; (3) the region contains a variant that impacts both trait 1 and trait 2; (4) 2 different variants that influence 2 traits separately are contained in the region. In this study, the genomic regions were predefined by independent blocks based on patterns of LD in the European populations as used in GSMR analysis. The software gwas-pw v0.21 (Pickrell et al., 2016) was used to calculate the posterior probability of each genomic region for each pair of immune and growth GWAS. In addition to *Z* scores, gwas-pw requires variance of effect size of each SNP. The allele frequencies of European ancestry individuals in the 1000G Project were therefore used to estimate the variance of effect size estimates. For pairs of growth and immune GWAS that shared overlapping samples, the genetic correlation between each pair of phenotypes calculated by LD score regression was offered to specify the expected correlation in summary statistics under the null. The genomic regions with posterior probabilities ≥0.9 were considered to be candidate regions containing variants influencing the pairs of traits simultaneously. Meanwhile, the SNPs involved in these candidate regions and had the highest *Z* scores for the 2 traits, respectively, were annotated functionally based on RefSeq transcripts using ANNOVAR (Wang et al., 2010).

### Identification of KEGG Pathways Shared Between Immune and Growth Traits

In order to capture the shared pathways between each pair of immune and growth traits that were beyond the spatial restriction of pleiotropy located on the sharing parts along the genome, enriched KEGG pathways were identified for each trait using GSA-SNP2 software (Yoon et al., 2018). This method is a powerful competitive pathway analysis tool that only requires the *P*-values of the SNPs in each GWAS. Compared with other methods, GSA-SNP2 can control type I error and maintain higher statistical power, and uses gene scores that indicate accurate pathway analysis results (Yoon et al., 2018). In this study, 218 KEGG pathways included in the GSA-SNP2 software were used as gene sets for enrichment analysis. GSA-SNP2 controls type I error via the SNP-count adjusted gene scores, and corrects for multiple-testing *P*-values by the false discovery rate (FDR). KEGG pathways with FDR ≤ 0.05 were considered to be significant. The overlapping significant pathways between each pair of immune and growth phenotypes were extracted, and the *P*-values for overlaps were calculated based on the empirical distributions using permutation with 1000 iterations.

## Results

### Correlation of Effect Sizes Between Growth and Immune Traits

**FIGURE 1:**
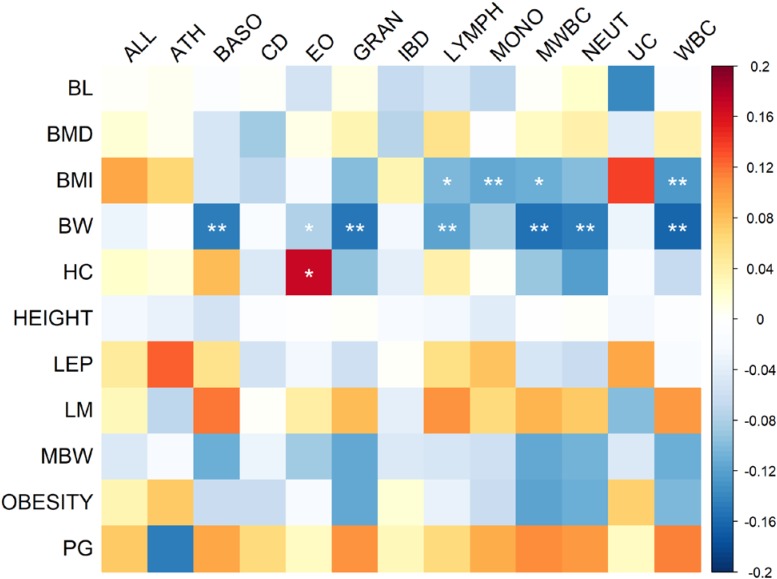
Heat map of genetic correlation coefficients between immune and growth phenotypes. The *P*-values of correlation coefficients were corrected by FDR. Genetic correlations with FDR less than 0.05 are indicated by one star, whereas correlations with FDR less than 0.01 are indicated by two stars.

*Z* scores of heritabilities for two growth traits, GWGM and GWGO that were less than 4 were excluded in the calculation of genetic correlation between growth and immune traits using cross-trait LD score regression. The pattern of genetic correlation coefficients is shown in **Figure 1**, and the values can be found in **Supplementary Table S1**. The *P*-values were corrected by FDR. The largest correlation coefficient (0.172) was between HC and eosinophil counts (EO). The most significant (FDR = 8.21 × 10^−7^) genetic correlation (-0.164) was between BW and white blood cell count (WBC). The significant correlation coefficients were predominately observed between BW, BMI, and several immune cell count phenotypes, and these correlation coefficients were all negative. ALL and ATH often had positive correlations with growth traits, although they were not statistically significant. This pattern was also true for pubertal growth (PG) and LM, which were positively correlated with immune phenotypes.

### Bi-directional Mendelian Randomization Between Immune and Growth Traits

**FIGURE 2:**
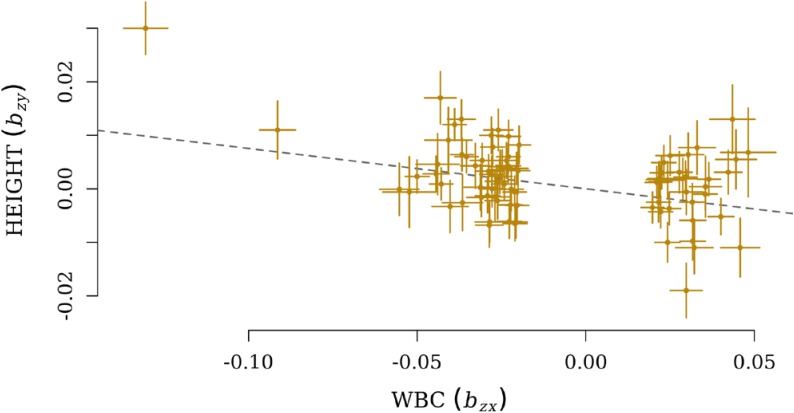
Plots of effect sizes of independent lead SNPs for WBC (*b_zx_*) on the *x*-axis and effect size for HEIGHT on the *y*-axis (*b_zy_*). The dotted line represents a line with a slope of (*b_xy_*).

After the filtration, GSMR calculated *b_xy_* for 206 pairs of exposure and outcome, and the Bonferroni cut-off for statistically significance was therefore set as 0.05/206. Finally, five pairs of exposure and outcome were significant for GSMR analysis with HEIDI-outlier correction (**Supplementary Table S2**). HEIGHT was the only growth-related phenotype involved, and it was influenced by four immune cell exposures: myeloid white blood count (MWBC), neutrophil count (NEUT), granulocyte count (GRAN), and WBC. Meanwhile, a significant effect from HEIGHT on ALL (*b_xy_* = −0.036, *SE* = 0.01, *P* = 1.45 × 10^−4^) was also observed. These causal relationships were all negative, and the most significant effect was observed from WBC on HEIGHT (*b_xy_* = −0.077, *SE* = 0.013, *P* = 2.27 × 10^−9^, **Figure 2**).

### Pleiotropic Variants Between Immune and Growth Traits

**FIGURE 3:**
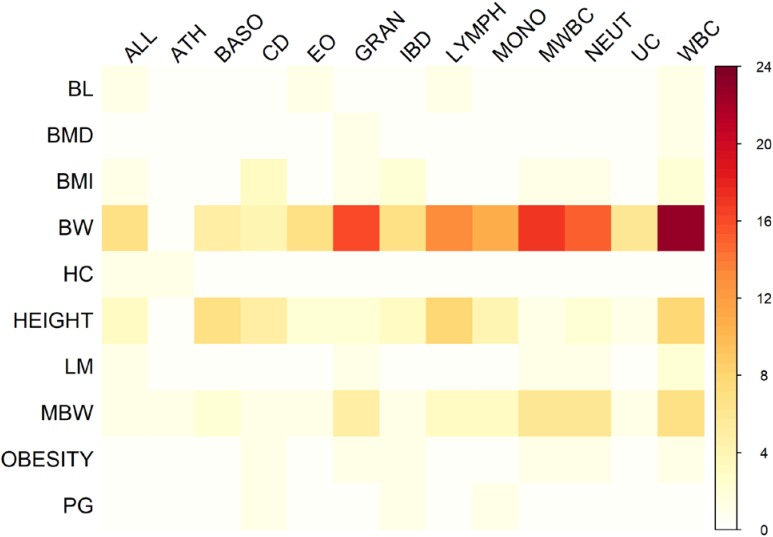
Heat map of the number of pleiotropic regions shared between immune and growth phenotypes.

**FIGURE 4:**
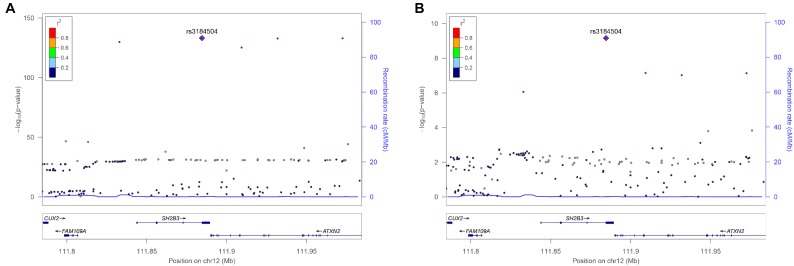
Regional association plot of a 100 kb window surrounding the pleiotropic SNP rs6569648 related to **(A)** HEIGHT and **(B)** LYMPH.

There were 98 genomic regions potentially containing variants related to immune and growth traits simultaneously (**Supplementary Table S3**). If a region was identified to be related to more than one pair of immune and growth GWAS, then it could be related to more than two phenotypes. Some regions might contain variants related to multiple phenotypes. For instance, a region (24.69–26.89 Mb) on chromosome 2 contained 5 SNPs related to three growth traits of childhood BMI (BMI), childhood obesity (OBESITY), pubertal growth (PG), and two immune diseases, CD and IBD (**Supplementary Table S3**). The causal genes might be *ADCY3* and *DNAJC27* (**Table 2**), which have previously been identified to be related to obesity (Stergiakouli et al., 2014) and pubertal growth (Cousminer et al., 2013), respectively. **Figure 3** shows the numbers of pleiotropic regions between pair-wise immune and growth phenotypes. The pleiotropic regions were more frequently observed between the growth traits BW, HEIGHT, and birthweight (maternal) (MBW), and the immune traits WBC, CD, and IBD (**Figure 3**). Some regions contained causal pleiotropic variants that had previously been identified to be pleiotropic (Pickrell et al., 2016). For instance, rs13107325 located in the exonic region of zinc transporter *SLC39A8* was related to ALL (*Z* = 4.51, *P* = 6.6 × 10^−6^), CD (*Z* = 7.06, *P* = 1.66 × 10^−12^), IBD (*Z* = 4.98, *P* = 6.44 × 10^−7^), BMI (*Z* = 5.70, *P* = 1.19 × 10^−8^), and HEIGHT (*Z* = −5.11, *P* = 3.3 × 10^−7^); rs1260326 located in the exonic region of *GCKR* was related to CD (*Z* = −6.54, *P* = 6.31 × 10^−11^), granulocyte count (GRAN) (*Z* = −8.62, *P* = 6.81 × 10^−18^), IBD (*Z* = −5.33, *P* = 9.61 × 10^−8^), lymphocyte counts (LYMPH) (*Z* = −7.03, *P* = 2.11 × 10^−12^), MWBC (*Z* = −7.83, *P* = 4.95 × 10^−15^), NEUT (*Z* = −9.07, *P* = 1.15 × 10^−19^), WBC (*Z* = −9.35, *P* = 8.75 × 10^−21^), and HEIGHT (*Z* = 6.76, *P* = 1.4 × 10^−11^). **Table 2** lists some potential genetic variants that are both significant for immune and growth traits in these regions and genes affected by these variants. Some pleiotropic variants had opposite Z statistics for immune and growth traits. For example, the SNP (rs3184504) located in the exon of *SH2B3* (**Figure 4** and **Table 2**) was significantly associated with MBW (*Z* = 6.168, *P* = 6.9 × 10^−10^) and LYMPH (*Z* = −24.624, *P* = 6.93 × 10^−134^).

**Table 2:** Pleiotropic SNPs influencing multiple immune and growth phenotypes.

SNP	Location	Gene	Traits
rs11676272	Exonic	*ADCY3*	CD, IBD, BMI, PG, OBESITY
rs1172294	UTR3	*DNAJC27*	CD, IBD, BMI, PG, OBESITY
rs1260326	Exonic	*GCKR*	CD, GRAN, IBD,LYMPH, MONO, MWBC, NEUT, WBC, HEIGHT
rs13107325	Exonic	*SLC39A8*	ALL, CD, IBD, BMI, HEIGHT
rs3184504	Exonic	*SH2B3*	ALL, BASO, CD, EO, GRAN, IBD, LYMPH, MONO, MWBC, NEUT, UC, WBC, BW, MBW

### KEGG Pathways Shared Between Immune and Growth Traits

**FIGURE 5:**
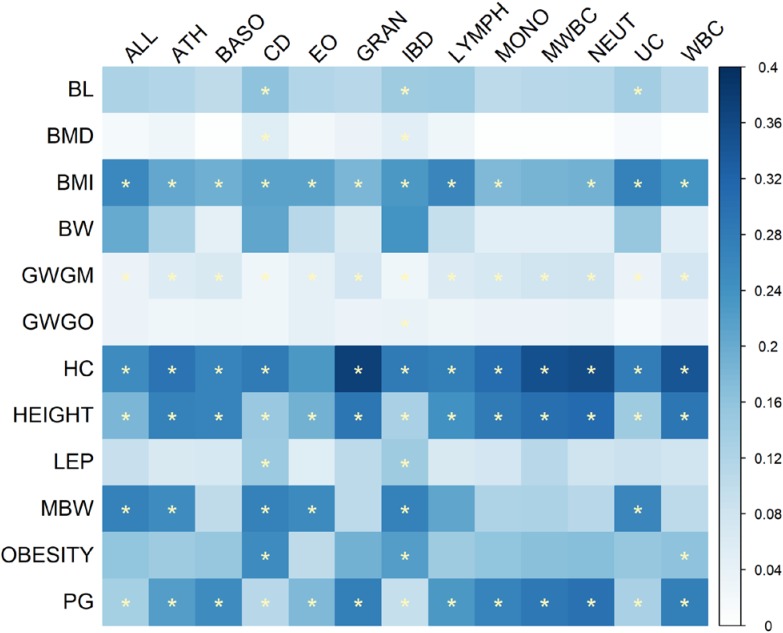
Heat map of the proportions of the number of overlapping KEGG pathways between immune and growth phenotypes accounting for the size of union set of KEGG pathways between pair-wise traits. The significant overlaps with Bonferroni-corrected *P*-values less than 0.05 are indicated by stars.

**Figure 5** shows the proportions of the number of overlapping KEGG pathways between immune and growth phenotypes accounting for the size of the union set of KEGG pathways between the pair-wise phenotypes. The significant overlaps were mainly observed between growth traits BMI, GWGM, HC, HEIGHT, and PG, and immune phenotypes. Although the relationship between BW and immune phenotypes was evident in the results of cross-trait LD score regression and pairwise GWAS pleiotropy mapping, no significant overlapping KEGG pathways could be identified between BW and immune traits. **Supplementary Table S4** lists the number of pathways that were included in significant overlaps between immune and growth traits. Many disease pathways are present in the top of the list, including the autoimmune diseases [systemic lupus erythematosus (hsa05322), graft-versus-host disease (hsa05332), autoimmune thyroid disease (hsa05320), and asthma (hsa05310)], as well as viral myocarditis (hsa05416) and type I diabetes mellitus (hsa04940). Some pathways were associated with immunity and growth simultaneously, such as the adipocytokine signaling pathway (hsa04920) (Ogunyemi et al., 2013; Procaccini et al., 2013) and *JAK*-*STAT* signaling pathway (hsa04630) (Wong and Fish, 2003).

## Discussion

### Summary Statistics of Immune and Growth Traits

Although the relationship between immune and growth traits has not been explored specifically in human genetics, their cryptic association has been observed and explained in other contexts. For instance, Urlacher et al. (2018) identified the negative effect of immune activity on growth in a sample of 261 Amazonian forager-horticulturalist Shuar children, because immune function is an energetically costly physiological activity that consumes calories that are needed for less immediately essential life activities such as growth (Urlacher et al., 2018). However, the aim of the current study was to explore their relationship in the context of genetics. Many growth and immune phenotypes were included as growth and immune traits encompass wide biological concepts. Growth is the enlargement of a tissue or organism; thus, the consequence of growth is not only size (HEIGHT, BL, HC, PG, BMD), but weight-related or obesity-related traits (BW, GWGO, GWGM, LM, MBW, OBESITY, BMI, LEP). Some of the relationships among growth traits were negative. For instance, BMI, a ratio trait defined by body weight divided by the square of height, was negatively related to HEIGHT from its definition. This is also the case for LEP, as the increase of LEP levels results in decrease of obesity. In contrast, the relationships among immune traits involved in this study were simple, for the reason that a high level of immune cells in serum is often a marker of autoimmune diseases.

### Genetic Correlation Between Immune and Growth Phenotypes

The result of cross-trait LD score regression (**Figure 1** and **Supplementary Table S1**) indicated significant negative correlations between BW and immune cell counts, but there appeared to be no correlation between BW and autoimmune diseases. The original study from which the summary statistics of BW were obtained also recorded a nearly zero correlation coefficient between BW and autoimmune diseases using cross-trait LD score regression (Horikoshi et al., 2016). It has also been confirmed that children with low birthweight are prone to have low immune capacity but higher levels of serum inflammation factors (Raqib et al., 2007). Low birthweight often results from fetal insult or nutritional insufficiency and manifests an increase in immune blood cells, which can cause allergic diseases such as asthma in child- or adulthood (Shaheen et al., 1999). In contrast, the most significant positive correlation was observed between HC and EO. HC appears to have some positive relationships with autoimmune diseases, which has been affirmed in a previous study (Eviston et al., 2015), where a positive correlation between childhood allergy and *in utero* head circumference was reported. Negative significant correlations were observed between BMI and immune cells (**Figure 1** and **Supplementary Table S1**), but the correlation coefficients between BMI and autoimmune diseases were all positive, although they were not significant. This inconsistent relationship might also help to explain the complex U-shaped pattern of the relationship between BMI and autoimmune diseases, suggesting that low and high BMI are both positively related to high risk of autoimmune diseases (Harpsøe et al., 2014). In addition, the significant genetic correlations were mainly between immune phenotypes and growth phenotypes measured in early age, such as BW, HC, and childhood BMI, indicating that early growth measurements may be suitable indicators for human immunity or allergic disease susceptibility in child- or adulthood. Even though the direction of these correlations varied in different pairs of phenotypes, obesity, low BW, and long HC were generally able to predict immunity problems.

Furthermore, in the absence of original information regarding the measurement of these traits, these genetic correlations might not be completely accurate simply based on summary statistics, and they should not be fully applied in other populations in different environments, as the different LD patterns and genotype by environmental interaction can cause variation in genetic correlation in different ethnic populations. In this study, we focused on the genetic correlation between immune and growth traits in European ancestry. A previous GWAS study performed in a Japanese population showed a significant positive genetic correlation between BMI and asthma, but a negative genetic correlation between BMI and rheumatoid arthritis. In addition, human height was negatively correlated with two autoimmune diseases, Graves’ disease and rheumatoid arthritis, although these correlations were not significant (Kanai et al., 2018). These results are generally consistent with the pattern of genetic correlation in our study, in which positive and negative correlations were both observed between BMI and some immune phenotypes, and negative but not significant correlations were observed between HEIGHT and immune phenotypes, indicating the accuracy of our results to some extent.

### Cause and Effect Relationship Between Immune and Growth Phenotypes

The GSMR results show that HEIGHT was negatively affected by different phenotypes of immune cell count. This is consistent with the energy allocation theory, which proposes that activation of immune system has a negative effect on growth (Rauw, 2012). Meanwhile, a unique significant effect of growth on immune phenotype was determined between HEIGHT and ALL. Previous studies have found that allergic diseases such as moderate or severe asthma can cause a delay in puberty stretch and affect final adult height (Hauspie et al., 1976, 1977; Preece et al., 1986; de Góes Antonio et al., 2003). The significant cause and effect between HEIGHT and ALL did not comport with these findings, indicating the complexity of their relationship. The results of GSMR were not consistent with the results that indicated no significant genetic correlation between HEIGHT and immune phenotypes. This might be because the cause and effect relationship between human height and immunity was explored by GWS SNPs shared between the two traits, while the genetic correlations were calculated using overlapping SNP effects across the whole genome. The statistical power of the GSMR analysis increases with the number of instrumental SNPs (Zhu et al., 2018). The small numbers of GWS SNPs for many growth phenotypes were not sufficient for GSMR (at least 10 independent GWS SNPs are required to perform the test); thus, except for HEIGHT, no significant causation relationship was identified for all other growth traits. In addition, with the HEIDI-outlier filtering pleiotropic SNPs, GSMR would further reduce the number of GWS SNPs used. HEIDI-outlier was used to filter SNPs that deviated from the hypothesis under the causal model that the expected values of estimated effects from exposure on outcome were identical for any instrumental SNP. Only five significant pairs were identified after HEIDI-outlier filtration, indicating that the link between growth and immunity might not fully be a cause and effect relationship. HEIDI-outlier was designed to reduce the inflation of GSMR, but not for identification of the true pleiotropic loci that have effects on multiple phenotypes simultaneously.

### Identification of Pleiotropic Variants

In this study, we used a hierarchical method to identify pleiotropic SNPs between pairs of immune and growth phenotypes. Methods such as *moloc* (Giambartolomei et al., 2018), which can identify pleiotropic loci for more than two traits, have high computational demand, and were thus not suitable given the fact that 26 phenotypes were involved in this study. In addition, gwas-pw has the potential to locate pleiotropic loci related to multiple traits. For instance, if the pairwise scan for phenotypes A and B, and phenotypes B and C were both indicated in the same region, it can be counted as those three phenotypes (A, B, and C) sharing an association in the same region (Pickrell et al., 2016). Some multiple-trait sharing regions were consistent with previous results, and some pleiotropic SNPs in this study were also detected in a previous study that explored pleiotropy among many different kinds of phenotypes (Pickrell et al., 2016), such as rs3184504 located in *SH2B3*, rs13107325 in *SLC39A8* gene and rs1260326 located in *GCKR*. The identification of the pleiotropy of these SNPs for immune and growth traits extended their functional spectrum in different traits. This also means that some pleiotropy among different traits can be explained by the same genetic variants (Visscher et al., 2017). In the era of precision medicine or genome editing, this pleiotropy indicates that it is not adequate to simply focus on a single phenotype, especially when the variants play opposite roles in various phenotypes (Parkes et al., 2013; Gratten and Visscher, 2016). However, for some quantitative phenotypes with polygenic backgrounds, pleiotropy mapping can be helpful not only in guiding drug development or genome editing to avoid loci with opposite functions on multiple phenotypes, but also to focus on the loci that contribute to the multiple phenotypes of interest simultaneously.

Several genes containing pleiotropic SNPs were indeed associated with growth and immune traits. For instance, *SH2B3* (**Figure 4**) is known to regulate cytokine and growth factor signals (Mori et al., 2014). Some pleiotropic findings were in causal genes, which support energy allocation theory for the relationship between immunity and growth. For instance, *ADCY3*, an obesity related gene, plays a role in energy homeostasis (Saeed et al., 2018). Meanwhile, it can catalyze the formation of cyclic AMP and regulate dendritic cells in the immune response (Chinn et al., 2016). In addition, gwas-pw could not distinguish a single causal variant that is pleiotropic (model 3) from 2 independent causal variants (model 4) if there existed strong LD between the two variants (Pickrell et al., 2016), although these variants could explain the genetic correlation between phenotypes in the context spurious pleiotropy (Solovieff et al., 2013).

### Mediated Pleiotropy Indicated by Sharing Pathways

Mediated pleiotropy is another type of pleiotropy (Solovieff et al., 2013) that describes different traits-related genes interacting with each other in pathways or networks (Zhang et al., 2016). The significantly shared pathways (**Figure 5**) helped complement the genetic explanations of the correlation between immune and growth traits. Although significant genetic correlations were observed between BW and immune traits, there was no pathway significantly shared between them, indicating that the genetic correlation between them primarily results from biological or spurious pleiotropy. The most frequent KEGG pathways included in significant overlaps between immune and growth phenotypes were associated with autoimmune diseases, such as systemic lupus erythematosus (hsa05322) (**Supplementary Table S4**). Some studies have suggested the relationship between obesity and autoimmune diseases (Harpsøe et al., 2014; Versini et al., 2014). Thus, as a risk factor, obesity might influence autoimmune disease through these pathways. In addition, the growth and immune functions can play roles in common diseases, as in the case of type I diabetes mellitus (hsa04940), which was affected by GH (Holly et al., 1988) and immune dysfunction (Geerlings and Hoepelman, 1999). These findings again indicate the importance of growth measurement for diagnosis of immunity-related diseases, and vice versa.

## Conclusion

In this study, we explored the genetic correlation between growth and immune phenotypes using summary statistics of a number of different GWAS. The results show that the directions of these correlations varied in different pairs of phenotypes. In addition, there was a negative cause and effect relationship between height and some phenotypes of immune cell count or allergic disease, which bolsters the energy allocation theory of the relationship between growth and immune traits. The identification of several pleiotropic variants, genomic regions, and pathways extend the pleiotropy of some SNPs and is helpful in our understanding of the genetic background of the relationship between growth and immune traits, and is meaningful for disease diagnosis and drug development.

### URLs

EGG, http://egg-consortium.org/; GIANT, http://portals.broadinstitute.org/collaboration/giant/index.php/; GWAS Catalog, http://www.ebi.ac.uk/gwas/downloads/summary-statistics; 1000 Genomes Project, http://www.1000genomes.org/; ldsc, https://github.com/bulik/ldsc/; LD score, https://data.broadinstitute.org/alkesgroup/LDSCORE/; GSMR, http://cnsgenomics.com/software/gsmr/; LD blocks, https://bitbucket.org/nygcresearch/ldetect-data; gwas-pw, https://github.com/joepickrell/gwas-pw; ANNOVAR, http://annovar.openbioinformatics.org/en/latest/; GSA-SNP2, https://sourceforge.net/projects/gsasnp2/.

## Author Contributions

YP and QW conceived and designed the whole study. ZZ, PM, QL, QX, HS, and BO performed the analysis. ZZ wrote the manuscript. All authors reviewed and approved the manuscript.

## Conflict of Interest Statement

The authors declare that the research was conducted in the absence of any commercial or financial relationships that could be construed as a potential conflict of interest.
